# Psychiatric comorbidities of Incarceration in a Patient with Gender Dysphoria: A Case Report

**DOI:** 10.1192/j.eurpsy.2022.1547

**Published:** 2022-09-01

**Authors:** G. Gill, M. Zachary, P. Korenis

**Affiliations:** Bronx Care health System, Psychiatry, Bronx, United States of America

**Keywords:** Gender Dysphoria, Incarceration, Psychiatric comorbidities

## Abstract

**Introduction:**

Mental health remains key comorbidity in the transgender population. There are more grave consequences on mental health if there is long-term incarceration history of a transgender person. 21% of transgender women are incarcerated in their lifetime, compared to <3% of the US general population. Incarcerated, transgender women are typically at risk for verbal, physical, and sexual assault that has been cross-sectionally linked to poor mental health in transgender patients. Childhood traumas and Adverse childhood experiences like sexual abuse may attribute to gender dysphoria as well as the externalizing and internalizing behaviors of the child in later part of life.

**Objectives:**

Better understand Gender Dysphoria and Incarceration.

**Methods:**

A case report and review of the literature.

**Results:**

X is a 56-year-old transgender female, admitted for Major Depressive disorder with Psychotic features, and substance abuse disorder. She was disoriented to person place, or time, believing she was at the “Federal Penitentiary.” She was also selectively mute and socially isolative as well as unable to perform ADL’s. She has an extensive legal history, which started in 1985 when she burglarized a pharmacy store for estrogen. Patient was started on Sertraline, Mirtazapine, and Risperidone. She was still socially withdrawn but was soon oriented to person place, and time and was able to complete her daily tasks.

**Conclusions:**

In this poster we discuss the challenges of managing an acute patient with extensive legal and substance abuse history, while also addressing the features of gender identity disorder and highlighting the difficult path of both the patient and physician in managing these challenges.

**Disclosure:**

No significant relationships.

